# Identification of novel key amino acids at the interface of the transmembrane domains of human BST-2 and HIV-1 Vpu

**DOI:** 10.1186/1742-4690-10-84

**Published:** 2013-08-06

**Authors:** Xiaojing Pang, Siqi Hu, Jian Li, Fengwen Xu, Shan Mei, Jinming Zhou, Shan Cen, Qi Jin, Fei Guo

**Affiliations:** 1 MOH Laboratory of Systems Biology of Pathogens, Institute of Pathogen Biology, Beijing 100730, P. R. China; 2 Institute of Medicinal Biotechnology, Chinese Academy of Medical Sciences & Peking Union Medical College, Beijing 100730, P. R. China

**Keywords:** Human immunodeficiency virus type 1, Vpu, BST-2, Bioluminescence resonance energy transfer assay, Molecular dynamics simulation

## Abstract

**Background:**

BST-2 (bone marrow stromal cell antigen 2) is an interferon-inducible protein that inhibits virus release by tethering viral particles to the cell surface. This antiviral activity of BST-2 is antagonized by HIV-1 accessory protein Vpu. Vpu physically interacts with BST-2 through their mutual transmembrane (TM) domains. In this study, we utilized the BRET assay and molecular dynamics (MD) simulation method to further characterize the interaction of BST-2 and Vpu.

**Results:**

Amino acids I34, L37, P40 and L41 in the TM domain of BST-2, and L11, A18 and W22 in the TM domain of Vpu were identified to be critical for the interaction between BST-2 and Vpu. The residues P40 in the TM domain of BST-2 and L11 in the TM domain of Vpu were shown, for the first time, to be important for their interaction. Furthermore, triple-amino-acid substitutions, 14–16 (AII to VAA) and 26–28 (IIE to AAA) in Vpu TM, not the single-residue mutation, profoundly disrupted BST-2/Vpu interaction. The results of MD simulation revealed significant conformational changes of the BST-2/Vpu complex as a result of mutating P40 of BST-2 and L11, 14–16 (AII to VAA) and 26–28 (IIE to AAA) of Vpu. In addition, disrupting the interaction between BST-2 and Vpu rendered BST-2 resistant to Vpu antagonization.

**Conclusions:**

Through use of the BRET assay, we identified novel key residues P40 in the TM domain of BST-2 and L11 in the TM domain of Vpu that are important for their interaction. These results add new insights into the molecular mechanism behind BST-2 antagonization by HIV-1 Vpu.

## Background

The bone marrow stromal antigen 2 (BST-2, also referred to as Tetherin, CD317 or HM1.24) blocks the release of human immunodeficiency virus type 1 (HIV-1) by directly tethering viral particles to the membrane of infected cells [1,2]. BST-2 is a type II integral membrane glycoprotein of unusual topology. It comprises a short N-terminal cytoplasmic tail (CT), followed by a single-pass transmembrane domain (TM), a disulfide-linked coiled-coil ectodomain (EC), and a C-terminal glycosylphosphatidylinositol (GPI) component that acts as second membrane anchor [3,4]. This unique membrane topology suggests that BST-2 directly tethers budding virions to the membrane of infected cells. Indeed, results of electron microscopy revealed that BST-2 is located between virus and cell membranes as well as between tethered virions [2,5-7]. Furthermore, BST-2 diminishes the infectivity of released HIV-1 particles [8]. BST-2 has also been shown to restrict viral release of other enveloped viruses, including other retroviruses, filoviruses, arenaviruses, orthomyxoviruses, gamma-herpesviruses and rhabdoviruses [9-13]. These results suggest that BST-2 is a general cellular restriction factor for enveloped viruses.

HIV-1 viral protein U (Vpu) serves as a BST-2 antagonist [2]. Vpu is an 81-amino acid, oligomeric type I trans-membrane protein encoded by an alternative open reading frame in the *env* gene [14]. Vpu mediates the removal of BST-2 from its site of action on the cell surface, although the exact mechanism by which Vpu affects the internalization, recycling, membrane transport, or degradation of BST-2 requires further study [4]. Phosphorylation of a pair of conserved serine residues (S52 and S56) in the cytoplasmic tail of Vpu is required for efficient Vpu-mediated degradation of BST-2 [15]. These phosphorylated serines residues are recognized by an F-box-containing ubiquitin ligase subunit, β-TrCP-2. Vpu thus recruits the multisubunit SCF-β-TrCP E3 ubiquitin ligase complex that causes ubiquitination and degradation of BST-2 [15-18]. Binding of Vpu to BST-2 through their transmembrane domains is crucial for the antagonization [16,19-22]. NMR studies have shown that BST-2 and Vpu directly contact between their transmembrane domains [16]. Studies have revealed that mutation in the transmembrane of either BST-2 (L22, L23, G25, I26, V30, I33, I34, I36, L37, L41, and T45) or Vpu (A14, A18 and W22) renders BST-2 resistant to Vpu [16,23-25].

Bioluminescence resonance energy transfer (BRET) assay has been used for real-time monitoring of protein-protein interactions in live cells [26]. The nonradiative (dipole-dipole) transfer of energy from donor enzyme to complementary acceptor fluorophore occurs after substrate oxidation. Donor to acceptor energy transfer and consequent emission from acceptor generally indicates less than 10 nm of separation of the two proteins, suggesting that the protein of interest are likely to be interacting with each other (directly or as part of a complex) [27]. Disruption or deficiency of interaction between donor and acceptor proteins results in decreased donor to acceptor energy transfer. BRET has been used to study a wide range of protein-protein interactions in bacterial, plant and mammalian cells [28-30]. This technique has been previously utilized to study the possible effects of small molecules on BST-2/Vpu interaction [31].

In this study, we utilized the BRET assay to identify the amino acids in the TM regions of human BST-2 and HIV-1 Vpu that are required for their interaction in live cells. Several amino acids on each transmembrane domain were found crucial for the interaction of BST-2 and Vpu (BST-2: I34, L37, P40 and L41; Vpu: L11, A18 and W22). In addition to some of these interaction sites that were reported in other studies [24,25], our data further showed that P40 in the TM domain of BST-2 and L11 in the TM domain of Vpu were also important for this interaction. Furthermore, we observed that triple-amino-acid mutants, 14–16 (AII to VAA) and 26–28 (IIE to AAA) in Vpu TM significantly affected the interaction, but not seen for single-residue mutation. The novel residues of P40A of BST-2 and L11A, 14–16 (AII to VAA) and 26–28 (IIE to AAA) of Vpu led to unstable complex compared to the wild-type (WT) as shown by the results of molecular dynamics (MD) simulation. Further studies showed that loss of interaction between the two proteins directly affected the Vpu-mediated BST-2 degradation and the inhibition of viral release.

## Results

### Rluc-BST-2 and Vm-EYFP are suitable for BRET assay

**Figure 1:**
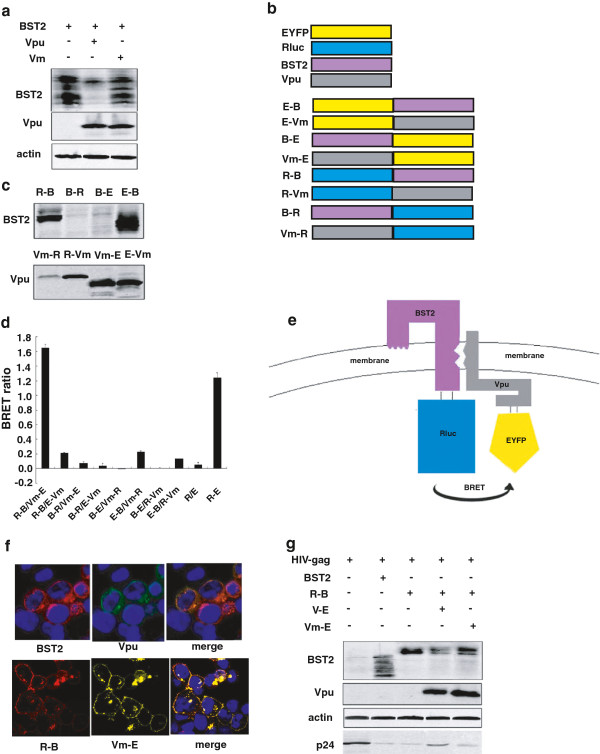
**BRET assay to monitor BST-2/Vpu interaction. (a)** Western blot analysis of cellular lysates following the cotransfection of BST-2 and Vpu or Vm plasmids into HEK 293 T cells. **(b)** Schematic representation of the plasmid DNA constructs. R-B and E-B were constructed with either Rluc or EYFP fused to the N terminus of BST-2; B-R and B-E were constructed with either Rluc or EYFP fused to the C terminus of BST-2; The N-terminus of Vm was fused with Rluc and EYFP to generate R-Vm and E-Vm; The C-terminus of Vpu was fused with Rluc and EYFP to engineer Vm-R and Vm-E. **(c)** Expression of the fusion proteins. Western blot analysis of the cellular lysates following the transfection of the expressing plasmid into HEK 293 T cells using anti-BST-2 and Vpu antibodies. **(d)** The BRET signals of BST-2 and Vpu interaction. HEK293T cells were transfected with different combinations of donor and acceptor DNA constructs. The BRET ratio was measured using a Pekin Elmer reader followed by the addition of substrate Coelenterazine at the final concentration of 5 μM. **(e)** The schematic diagram of the energy transfer between R-B and V-E. **(f)** Subcellular localization of R-B and Vm-E. Immunofluorescence staining was performed to detect the localization of BST-2(red), Vpu (green), R-B(red) and Vm-E(yellow) expressed in HEK 293 T cells. The nuclei were stained with DAPI (blue). Representative images are shown. **(g)** Function analysis of the tagged BST-2 and Vpu proteins. Western blot analysis of the cellular lysates and the corresponding viral lysates following cotransfection of the HIV-1 Gag-Pol construct (containing gag and pol genes) and other expressing plasmids into HEK293T cells using anti-BST-2, anti-Vpu, anti-actin and anti-p24 antibodies.

BRET signal was generated using Rluc (Renilla luciferase) and EYFP (Enhanced Yellow Fluorescent Protein) in this study. Rluc or EYFP was fused with human BST-2 or HIV-1 Vpu, respectively. In order to rule out the effect of BST-2 degradation by Vpu, the S52, 56A mutation of Vpu was used in the BRET study (Figure 1a). This is a degradation-null mutant but still interacts with BST-2, and is hereafter referred to as Vm. Eight plasmids were generated, namely Rluc-BST2 (R-B), EYFP-BST2 (E-B), BST2-Rluc (B-R), BST2-EYFP(B-E), Rluc-Vm(R-Vm), EYFP-Vm(E-Vm), Vm-Rluc(Vm-R), Vm-EYFP(Vm-E) (Figure 1b). Most of the fusion proteins were expressed well in transfected HEK 293 T cells except for B-E, B-R and Vm-R that had lower expression levels (Figure 1c). Every pair of fusion proteins, containing BST-2 and Vm fused with Rluc or EYFP, were co-transfected into HEK293T cells. R-B and Vm-E produced BRET signal as strong as the positive control R-E (Rluc fused with EYFP directly) (Figure 1d). We therefore used the R-B/Vm-E pair in the subsequent BRET assay to study BST-2 and Vpu interaction (Figure 1e). We next investigated the subcellular localization of the wild-type and mutated proteins. The results in Figure 1f revealed that R-B and Vm-E had localization similar to wild-type proteins. R-B and Vm-E were expressed at levels similar to the untagged, wild-type BST-2 and Vpu. HIV-1 VLP release was inhibited by R-B. In addition, V-E induced the degradation of R-B, and enhanced HIV-1 VLP release,while Vm-E neither degraded R-B nor rescued HIV-1 VLP release (Figure 1g).

### Use BRET assay to monitor the interaction of BST-2 and Vpu in live cells

**Figure 2:**
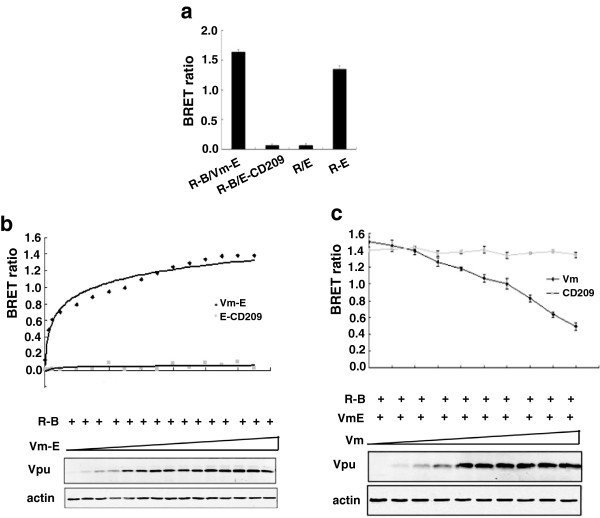
**The specificity of the BRET assay in detecting the interaction of BST-2 and HIV-1 Vpu. (a)** The specificity of BRET signals between BST-2 and Vpu. HEK293T cells were transfected with plasmid DNA and seeded into 96-well plates, and then the BRET ratio was measured by using a Pekin Elmer reader. **(b)** The saturation curve of the BRET assay. HEK293T cells were cotransfected with constant amount of R-B plasmid and increasing dose of Vm-E or E-CD209 plasmid. The BRET ratio was detected by using a Pekin Elmer reader. Western blot analysis of the cellular lysates was shown. **(c)** The competition curve of the BRET assay. HEK293T cells were cotransfected with constant amount of R-B plasmid and Vm-E and increasing dose of Vm or CD209 plasmid, and the BRET ratio was detected by using a Pekin Elmer reader. Western blot analysis of the cellular lysates was shown.

In order to determine the specificity of the interaction between BST-2 and Vpu, we substituted Vm with unrelated protein CD209 which is a type II transmembrane protein consisting of C-terminal extracellular domain, transmembrane region and N-terminal cytoplasmic region to perform BRET assay. The BRET signal was as low as the negative control R/E (Figure 2a). We further performed saturation and competition studies to validate the specificity of the BRET assay [26]. HEK293T cells were co-transfected with fixed amounts of plasmid expressing Rluc fusion protein R-B and increasing amounts of plasmid expressing EYFP fusion protein Vm-E. The saturation curve was generated, which showed that the BRET signal reached a saturation level with high concentrations of Vm-E. This phenomenon was not observed in cells co-expressing E-CD209 protein and R-B (Figure 2b). In addition, constant amounts of R-B and Vm-E proteins were co-expressed with untagged Vm that competed for interaction with the tagged protein Vm-E. A reduction of the BRET ratio was observed. Increasing the amounts of untagged Vm diminished the BRET ratio in a dose-dependent manner. Again, this phenomenon was not observed in cells co-expressing untagged protein CD209 that did not interact specifically with R-B (Figure 2c). These results indicate that the BRET assay is a useful and sensitive approach to study BST-2 and Vpu interaction in live cells.

### Identify the amino acids in the transmembrane domains of human BST-2 and HIV-1 Vpu critical for their interaction

**Figure 3:**
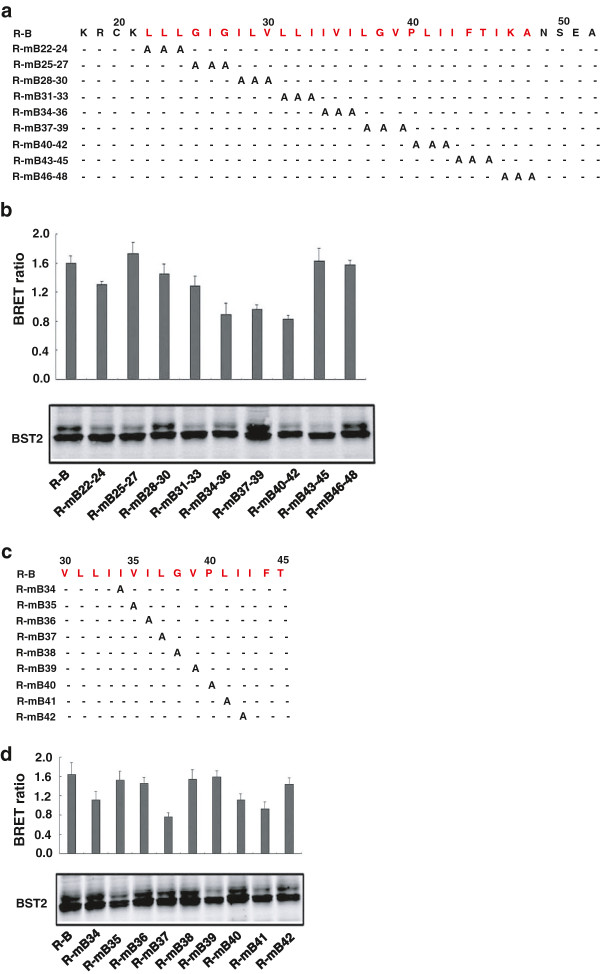
**The amino acids in TMD of human BST-2 responsible for HIV-1 Vpu interaction. (a)** Mutation of the R-B TM domain. Every three amino acids are mutated as a group. **(b)** HEK293T cells were cotransfected with Vm-E plasmid and R-B TM mutants, and the BRET ratio was detected by using a Pekin Elmer reader. Western blot analysis of the cellular lysates was shown. **(c)** Mutation of R-B TM domain. **(d)** HEK293T cells were cotransfected with Vm-E plasmid and a series of the R-B TM single amino acid mutants, and the BRET ratio was detected by using a Pekin Elmer reader. Western blot analysis of the cellular lysates was shown.

Previous studies have demonstrated that the interaction between human BST-2 and HIV-1 Vpu is mediated through their TM domains [20,21]. Further detailed mutagenesis studies revealed that multiple amino acid residues across the TM domains govern the interaction between human BST-2 and HIV-1 Vpu [23-25]. In this study, comprehensive mutagenesis and BRET assay were performed to further identify residues in TM domains of BST-2 and Vpu that are important for their interaction. Plasmids encoding R-B mutants with triple-alanine substitutions in TM domain of BST-2 (amino acids 22–48) were constructed. These mutants were expressed at levels comparable to the wild type (Figure 3a and 3b). We then transfected these mutants together with Vm-E plasmid into HEK293T cells. Forty-eight hours post-transfection, BRET assay was performed to determine the interaction between R-B and Vm-E. The R-mB_34-36_, R-mB_37-39_, R-mB_40-42_ mutants had significantly reduced BRET signal compared to that of wild type R-B (Figure 3b), indicating that the amino acids from position 34 to 42 were crucial for BST-2 and Vpu interaction in live cells. Nine single-alanine-substituted mutants from position 34 to 42 were constructed to further identify the key amino acids (Figure 3c). These nine mutants were expressed at wild type levels (Figure 3d). Mutation of residues I34, L37, P40 and L41 in TM domain of BST-2 reduced BRET signals. A novel residue P40 was shown for the first time being critical for BST-2 and Vpu interaction.

**Figure 4:**
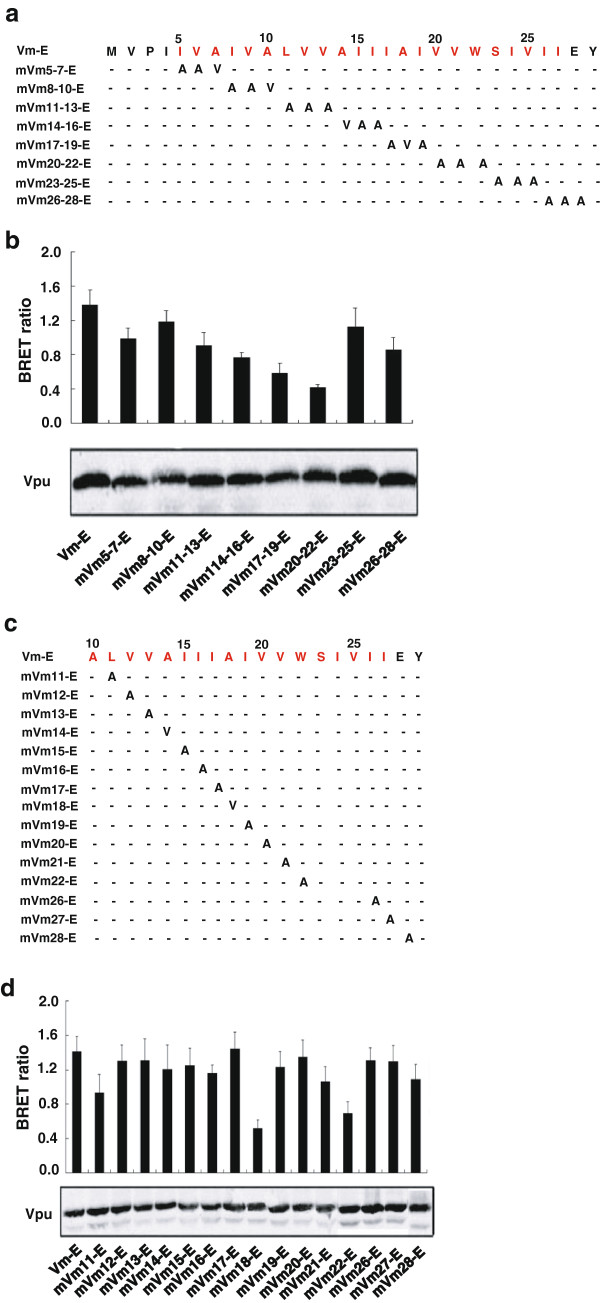
**The amino acids in TMD of HIV-1 Vpu responsible for human BST-2 interaction. (a)** Mutation of the Vm-E TM domain. Every three amino acids were mutated as a group. **(b)** HEK293T cells were cotransfected with R-B plasmid and a series of Vm-E TM mutants, and the BRET ratio was detected by using a Pekin Elmer reader. Western blot analysis of the cellular lysates was shown. **(c)** Mutation of the R-B TM domain. **(d)** HEK293T cells were cotransfected with R-B plasmid and a series of Vm-E TM single amino acid mutants, and the BRET ratio was detected by using a Pekin Elmer reader. Western blot analysis of the cellular lysates was shown.

In order to identify the residues in Vpu that are important for binding to BST-2, plasmids encoding Vm-E mutants with triple-amino acid substitutions in TMD of Vpu (amino acids 5–27) were constructed with non-alanine to alanine residues and alanine to the same hydrophobic valine residues (Figure 4a). All mutants were expressed well in transfected HEK293T cells (Figure 4b). Among the eight triple-amino acids-substituted mutants, mVm_11-13_-E, mVm_14-16_-E, mVm_17-19_-E, mVm_20-22_-E, mVm_26-28_-E had markedly reduced BRET signal (Figure 4b). In order to determine the specific amino acids responsible for the interaction between Vpu and BST-2, each amino acid in Vm-E from positions 11 to 22 and 26 to 28 was mutated with non-alanine to alanine residues and alanine to valine residues (Figure 4c). All mutants had the same expression levels compared with Vm-E (Figure 4d). The mVm_18_-E and mVm_22_-E mutants showed significantly decreased BRET ratio, and mVm_11_-E mutant had decreased BRET ratio to a lesser extent (Figure 4d). Unexpectedly, some triple substitutions in the Vm TMD had significantly decreased BRET signal, such as mVm_14-16_-E and mVm_26-28_-E, but none of a single mutation of the three residues generated such an effect.

### Effect of TM mutations on BST-2/Vpu interaction by molecular dynamics simulation

**Figure 5:**
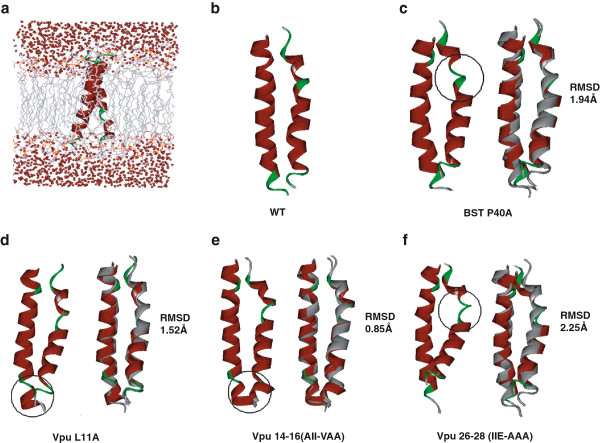
**Structures of BST-2/Vpu complex determined by molecular dynamics simulations.** The representative structure was generated by cluster analysis, and the alignment of the mutant complexes (in ribbon, colored by secondary structure, helixes are in red, and turns or loops are in green) with the wild-type complex (gray ribbons) was conducted in DS **(c-f)**. The significant conformational changes between the WT and mutants are highlighted by the black circles. **(a)** Description of the simulation model in membrane; **(b)** Representative structures of the complex of the wild type (WT) BST-TM and Vpu-TM; **(c)** Effect of the P40A mutation in BST2-TM on the BST-2 − Vpu interaction. **(d)** Effect of the L11A mutation in Vpu-TM on the BST-2 − Vpu interaction. **(e)** Effect of the 14–16 (AII to VAA) mutation in Vpu-TM on the BST-2 − Vpu interaction. **(f)** Effect of the 26–28 (IIE to AAA) mutation in Vpu-TM on the BST-2 − Vpu interaction.

We further investigated how the mutants that were first identified in our study affect the interaction between BST-2 and Vpu by performing molecular dynamics (MD) simulation. The simulation models were shown in Figure 5a. Cluster analysis was also performed to generate the representative complex structure of the largest family during the simulation. For all the mutant complexes, the conformation of Vpu-TM fits to that of the WT complex very well (Figure 5b). Thus, we aligned the representative structure of the mutant complex with the WT complex through fitting the Vpu-TM structure, and the results are shown in Figure 5c-f. Briefly, compared to the wild type (WT) complex, the mutants showed less stable complexes which were similar to previous simulation results of the complex with I34, L37, or L41 in the TM domain of BST-2, as well as A14, A18 or W22 in the TM domain of Vpu [32]. To evaluate the overall conformational change of mutant complexes, the fitting RMSD values were calculated for each mutant complex through the alignment with the WT complex. The RMSD values of P40A mutant at BST-2, L11A mutant, 14–16 (AII to VAA) mutant, or 26–28 (IIE to AAA) mutant at Vpu-TM are 1.94 Å, 1.52 Å, 0.85 Å, or 2.25 Å respectively, which means a close overall conformation comparing to the WT complex. However, a destruction of helix at 38–41 position of BST2-TM was observed for all mutants as a result of simulation. P40A mutant in BST-2 (Figure 5c) or 26–28 (IIE to AAA) mutant in Vpu (Figure 5f) cause more significant conformational change than the other two mutants. Also, L11A mutant or 14–16 (AII to VAA) mutant in Vpu-TM destabilizes the helix conformation of 23–25 motif in Vpu but reconstructs the helix structure of 26–30 motif (Figure 5d, 5e). Thus, marked local conformation changes are seen for all four mutants, and such conformational changes likely disturb the interaction between BST-2 and Vpu.

### Functional validation of the identified key residues in the TM domains of human BST-2 and HIV-1 Vpu

**Figure 6:**
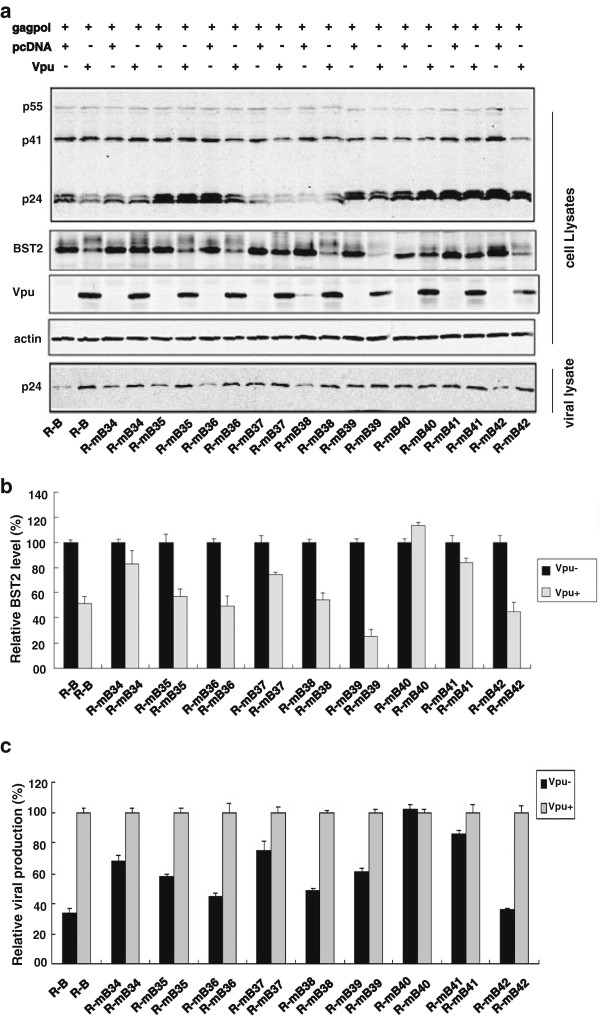
**Functional validation of the crucial residues in the TMD of BST-2 for Vpu interaction. (a)** Western blot analysis of the cellular lysates and the corresponding viral lysates following the cotransfection of the HIV-1 Gag-Pol construct (containing gag and pol genes) and other plasmids into HEK293T cells. **(b)** Relative BST-2 reduction was determined by densitometric scanning using the ImageJ program (NIH) aiming at the specific bands. BST-2 level in cells that were cotransfected with R-B or R-B mutants and together with pcDNA was arbitrarily set as 100%. The data shown was the average of three independent experiments. **(c)** Relative HIV-1 VLPs release was assessed by densitometric scanning using the ImageJ program (NIH). The level of HIV-1 VLPs released from cells that were cotransfected of the HIV-1 Gag-Pol construct and R-B or R-B mutants and together with pcDNA was arbitrarily set as 100%. The data shown was the average of three independent experiments.

To verify the functional role of the identified residues in TM domain of BST-2, we tested these BST-2 mutants for their sensitivity to Vpu. HEK293T cells were cotransfected with HIV-1 Gag-Pol construct (containing gag and pol genes) and Vpu together with a WT R-B or R-B mutants. The virus-like particles (VLP) in the culture supernatants together with proteins expressed in cell extract were assessed by Western blotting. As shown in Figure 6a and 6c, the mutants the mutants R-mB_34_, R-mB_37_, R-mB_40_ and R-mB_41_ became partially or completely resistant to Vpu antagonization. To determine whether mutants resistant to Vpu antagonization are also resistant to the degradation by Vpu, BST-2 in cellular lysates was examined by Western blotting. The results showed that R-mB_34_, R-mB_37_, R-mB_40_ and R-mB_41_ were not degraded by Vpu as much as wild type BST-2 (Figure 6a, 6b). In order to rule out the possible effect of the Rluc sequence on the ability of BST-2 to inhibit virus release, we also inserted the above mutations into the wild type BST-2 and observed similar results (Additional file 1: Figure S1a). These data provided direct evidence that I34, L37, P40 and L41 of human BST-2 determine Vpu susceptibility in addition to Vpu interaction. The loss of interaction between the two proteins directly affected the subsequent BST-2 degradation and the inhibition of viral release (Figure 6a, 6c).

**Figure 7:**
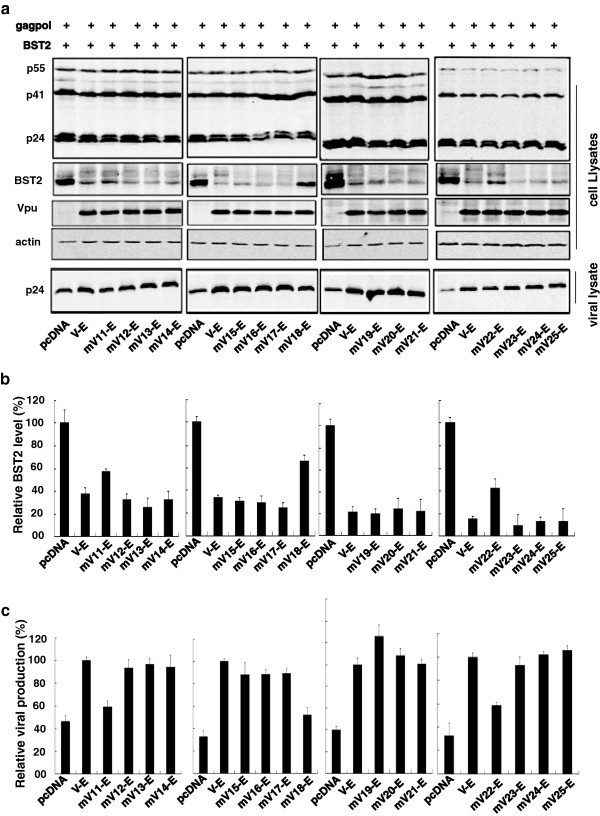
**Functional validation of the crucial residues in the TMD of Vpu for BST-2 interaction. (a)** Western blot analysis of the cellular lysates and the corresponding viral lysates following the cotransfection of the HIV-1 Gag-Pol construct (containing gag and pol genes) and other expressing plasmids into HEK293T cells. **(b)** Relative BST-2 reduction was determined by densitometric scanning using the ImageJ program (NIH). BST-2 level in cells that were cotransfected with HIV-1 Gag-Pol, R-B and pcDNA was arbitrarily set as 100%. The data shown were the average of three independent experiments. **(c)** Relative HIV-1 VLPs release was assessed by densitometric scanning using the ImageJ program (NIH). The level of HIV-1 VLPs released from cells that were transfected with HIV-1 Gag-Pol, R-B and V-E was arbitrarily set as 100%. The data shown were the average of three independent experiments.

To further characterize the function of the residues in Vpu TMD, a series of Vpu TM crucial mutants were constructed containing the wild type S52 and S56 residues. HEK293T cells were cotransfected with HIV-1 Gag-Pol construct and BST-2 together with a series of key amino acid substituted V-E TM mutants. The VLP in the culture supernatants together with proteins expressed in cell extract were determined by Western blotting. The results showed that mutants mV_11_-E, mV_18_-E, mV_22_-E partially lost their ability to promote HIV-1 VLP release (Figure 7a, 7c). In the meantime, mV_11_-E, mV_18_-E, mV_22_-E were unable to degrade BST-2 (Figure 7a, 7b). We also tested the above Vpu mutations by inserting them into the wild type Vpu and observed similar results (Additional file 1: Figure S1b). These results indicated that the L11, A18 and W22 residues in Vpu TM domain are essential for Vpu to counteract human BST-2 (Figure 7a, 7c).

## Discussion

BRET signal is a ratio of the EYFP long wave emission (530 nm) divided by the Rluc short wave emission (475 nm) [27]. The BRET based assay was for the first time used by Kuhl et al. to monitor BST-2/Vpu interaction with the aim to study the effect of small molecules in this regard [31]. A similar flow cytometry-based FRET assay was used by Schildler et al. to identify novel protein interaction partners in a high-throughput format [33]. Here we used BRET assay to identify the crucial amino acids in TM of the two proteins. In our study, Vm (S52, 56A mutant) were utilized instead of wild type Vpu because Vpu degrades BST-2 and this will complicate the study of Vpu/BST-2 interaction. After testing different combinations of fusion proteins in BRET assay, we selected the group of BST-2 fused with Rluc at N-terminal and Vm fused with EYFP at C-terminal. Previous studies showed that tagging human BST-2 at N-terminus and tagging Vpu at C-terminus do not interfere with their function and cellular localization [24].

This technique allowed us to successfully identify four amino acid residues in human BST-2 TM (I34, L37, P40 and L41) and three amino acid residues in Vpu TM (L11, A18 and W22) that are required for their interaction. Findings with I34, L37 and L41 in BST-2 TM and A14, A18 and W22 in Vpu TM were reported by other groups [24,25]. However, the other two sites, P40 in BST-2 TM and L11 in Vpu TM were shown for the first time in this study as the critical determinants for Vpu/BST-2 interaction. It was reported that P40A mutation abolished susceptibility to Vpu without affecting the interaction of BST-2 and Vpu [24]. In contrast, our results showed that the P40A mutation affected this interaction to the same degree as I34A in our BRET assay. This discrepancy was likely due to the different approaches that were used to characterize BST-2 and Vpu interaction. As opposed to the BRET assay that we used, the BiFC method was employed in their study to monitor BST2 and Vpu interaction. In addition, a recent study based on cyteine cross-linking support the interaction between BST-2 P40C and Vpu A7C [34]. In addition, we replaced alanine in Vpu TM with valine and did not observe the effects of A14L and A14F as previously reported, but the A18V effect is in line with that shown for A18L and A18F [25,35]. However, the triple-amino-acids substitution of 14–16 (AII to VAA) produced significantly decreased BRET ratio. The same result was also seen in the triple-amino-acids substitution of 26–28 (IIE to AAA). Namely, substitution of multiple amino acids, but not a single residue mutation, significantly affects the interaction. It was reported that deletion of residues I15, I16 and I17 or deletion of I26 and I27 in Vpu TM potently block the physical interaction between human BST-2 and Vpu, and prevent Vpu from enhancing HIV-1 virus release [36]. These results indicated that multiple isoleucine in HIV-1 Vpu TM play an important role in forming the hydrophobic interaction surface between the two proteins. Different from other reports, we observed that L11 in Vpu TM also contributes to the interaction between the two proteins albeit to a lesser extent.

In summary, results of our BRET assays identified novel residues in the TM domains in human BST-2 and HIV-1 Vpu TM that are important for their interaction. Mutations P40A in BST-2 and L11A, 14–16 (AII to VAA) and 26–28 (IIE to AAA) were first shown in our study to interfere with the interaction of Vpu and BST-2. Results of molecular dynamics simulation further support this observation. Compared to the wild type complexes, the mutant complexes are less stable and show local conformation changes including the loss of helical structure and the formation of new helix. Such conformational changes possibly lead to the weakened interaction between BST-2 and Vpu. Further studies showed that the identified key residues in the TM domains of human BST-2 and HIV-1 Vpu were important for their respective functions. The loss of interaction between the two proteins directly affected the Vpu-mediated BST-2 degradation and the BST-2 inhibition of viral release.

BST-2/Vpu interaction has been used as the target to identify small molecules that antagonize HIV-1 Vpu function by cell-based ELISA monitoring the up-regulation of cell surface BST-2 [37]. BRET assay is simple, rapid, non-invasive, and is suitable for developing a new high-throughput screening assay. The screening assay based on BRET method can lead to discovery of small molecules that disrupt the interaction between BST-2 and Vpu and rescue the antiviral activity of BST-2.

## Conclusions

In this study, we utilized the BRET assay to further define the interaction of BST-2 and Vpu, and identified novel residues P40 in the TM domain of BST-2 and L11 in the TM domain of Vpu that were important for their interaction. Furthermore, triple-amino-acid substitutions, 14–16 (AII to VAA) and 26–28 (IIE to AAA) in Vpu TM, not single-residue mutation, exerted significant effect on BST2/Vpu interaction. The significant conformational changes of the BST2-Vpu complex structure caused by mutating P40 of BST-2 and L11, 14–16 (AII to VAA) and 26–28 (IIE to AAA) of Vpu were demonstrated by molecular dynamics (MD) simulation. In addition, disrupting interaction between the two proteins directly affected the subsequent BST-2 degradation and the inhibition of viral release. These results add new insights into the molecular mechanism behind BST-2 antagonization by HIV-1 Vpu.

## Methods

### Plasmids construction

The human BST-2 (hBST-2) and HIV-1 Vpu cDNA were gifts from Chen Liang (Lady Davis Institute for Medical Research, McGill University). pR-B, pE-B, pB-R, pB-E, pR-Vm, pE-Vm, pVm-R were generated by subcloning *Renilla* luciferase or EYFP cDNA and human BST-2 or Vpu cDNA into the pcDNA™3.1/V5-His A vector (Invitrogen). pVm-E was generated by subcloning codon optimization Vm cDNA into pEYFP-N1 (Invitrogen). A series of BST-2 and Vpu TM mutants were generated by overlapping PCR-based mutagenesis. pE-CD209 was generated by subcloning EYFP cDNA and CD209 cDNA into the pcDNA™3.1/V5-His A vector (Invitrogen). All constructs were verified by nucleotide sequencing. The Vpu, hBST-2 and p24 antisera were obtained from the NIH AIDS Research and Reference Reagent Program. PEI (408727) was purchased from Sigma. Coelenterazine h was purchased from Promega.

### Cell culture and transfection

Human embryonic kidney (HEK293T, ATCC #CRL-11268) cells were cultured in complete DMEM (Gibco) supplemented with 10% fetal calf serum (FCS), penicillin (100 U/ml), and streptomycin (100 μg/ml). 8 × 10^5^ cells/well was seeded in 6-well plates one day prior to transfection. Cells were transfected with various plasmids depending on the experiment, using PEI (Sigma) according to the manufacturer's protocol.

### Immunofluorescence staining and confocal microscopy

For immunostaining, cells on coverslips were transfected as described above and then fixed in 4% paraformaldehyde. After two washes in 1× phosphate buffered saline, the cells were permeabilized with 0.5% Triton X-100. Staining was performed with primary antibodies including Rabbit BST-2 antiserum (1:1,000 dilution) and Vpu (1:1,000 dilution). Anti-rabbit FITC-conjugated or TRITC-conjugated antibodies (1:500 dilution) were used as secondary antibodies. Images were recorded with a Leica TCS SPE, DM2500 confocal microscope (Leica Microsystems).

### BRET assay

HEK293T cells were seeded in six-well plates (8 × 10^5^ cells per well) 24 hours prior to transfection. Plasmids were transfected using PEI (Sigma) according to the manufacturer’s instructions. Forty-eight hours after transfection, cells were washed twice and detached into 1 ml 1× phosphate buffered saline, and then distributed into 96-well microplates at 1 × 10^5^ cells per well (Corning 3600). The substrate coelenterazine h (Promega) was added at a final concentration of 5 μM. Readings at 475 nm (reflecting the bioluminescence given off by Rluc) and 530 nm (reflecting the resonance energy transfer from Rluc to EYFP) were measured simultaneously by a Perkin Elmer instrument. The BRET ratio was calculated as emission at 530 nm (light emitted by EYFP)/emission at 475 nm (light emitted by Rluc). The BRET ratios reported were corrected by subtracting the ratios obtained in cells expressing the donor only (Rluc).

### Viral isolation

To produce viruses, cells were seeded in 10-cm dishes (5 × 10^6^ cells per dish) 24 hours prior to transfection. The HIV-1 Gag-Pol construct (containing gag and pol genes) and other plasmids were transfected using PEI (Sigma) according to the manufacturer’s instructions. Forty-eight hours after transfection, the culture supernatants were filtered through a 0.45 μm filter, and virus particles were pelleted by ultracentrifugation through a 20% sucrose cushion at 35,000 rpm for 1 hour at 4°C using a Beckman SW 41Ti rotor.

### Western blotting

Cells or viruses were lysed in RIPA buffer (0.1% SDS, 1% Triton X-100, 1% sodium deoxycholate, 150 mM NaCl, 10 mM Tris [pH 7.5], 1 mM EDTA), and equal amounts of cell or viral lysate were separated by SDS-PAGE on a 12% gel, followed by transferring proteins onto nitrocellulose membranes (Gelman Science). The membranes were probed with BST-2 antiserum (1:2,000 dilution), anti-Vpu serum (1:5,000 dilution), anti-p24 antibody (1:5,000 dilution), or a mouse monoclonal antibody against actin (1:5,000 dilution), followed by incubation with IRDye™ secondary antibodies (1:20,000). Protein bands were visualized with an LI-COR Odyssey instrument and quantified using the Image J automated digitizing program (NIH).

### Construction of initial model and MD simulations of BST-2 TM and Vpu TM complex

The initial complex structures for BST-2 TMD peptide and Vpu TMD peptide were generated using Discovery Studio 2.5 based the previous reported WT complex. The initial structures were embedded into an explicit DOPC lipid membrane including explicit solvent using Discovery Studio 2.5 to generate the input structures for the later molecular dynamic simulation in membrane. The membrane was generated using the VMD soft suit (*25*), following by the 3 ns pre-equilibrium in AMBER (University of California, San Francisco). The partial charges on DOPC monomer were calculated by fully reproducible ab-initio electronic structure computations with Gaussian 09 (Gaussian, Inc., Wallingford CT) using Hartree-Fock self consistent field (HF-SCF) with 6-31G* basis set after optimizing with DFT-B3LYP and successively with HF-SCF at 6-31G∗ level. The partial charges were then extracted following RESP protocol with ANTECHAMBER program of the AMBER suite. MD simulations were performed using the AMBER10 package, all-atom parm94 force fields, particle mesh-Ewald summation, SHAKE algorithm, and a time-step of 2 fs. The systems were minimized using the steepest decent method, followed by the conjugate gradient method to remove the bad contacts. An equilibrium period of 120 ps was performed before the MD simulations commenced. The 10 ns long MD simulations were conducted in the NPT ensemble at 310 K. After the simulation was completed, a cluster analysis was performed for the snapshots taken from the period of 3–9 ns, which amounts to 6000 snapshots for each simulation. Conformation clustering was performed with a radius of 2 Å using the “Kclust” module implemented in the MMTSB software suite (The Scripps Research Institute). The obtained representative structure of the largest cluster family would be served as the representative conformation for the corresponding complex.

## Competing interests

The authors declare that they have no competing interests.

## Authors’ contributions

FG, QJ, XP and SH conceived and designed the study. XP, SH, JL, FX, SM, and JZ carried out the experiments. SC, QJ and FG analyzed the data. FG, XP and SH drafted the manuscript. All authors read and approved the final manuscript.

## Supplementary Material

Additional file 1: Figure S1 Functional validation of the crucial residues in the TMD of BST-2 and Vpu for their interaction (a) Crucial residues in the TMD of BST-2 for Vpu interaction. Upper panel: Western blot analysis of the cellular lysates and the corresponding viral lysates following the cotransfection of the HIV-1 Gag-Pol construct and other plasmids into HEK293T cells. Middle panel: Relative BST-2 reduction was determined by densitometric scanning using the ImageJ program (NIH) aiming at the specific bands. BST-2 level in cells that were cotransfected with BST-2 or BST-2 mutants and together with pcDNA was arbitrarily set as 100%. Lower Panel: Relative HIV-1 VLPs release was assessed by densitometric scanning using the ImageJ program (NIH). The level of HIV-1 VLPs released from cells that were cotransfected of the HIV-1 Gag-Pol construct and BST-2 or BST-2 mutants and together with pcDNA was arbitrarily set as 100%. (b) Crucial residues in the TMD of Vpu for BST-2 interaction. Upper panel: Western blot analysis of the cellular lysates and the corresponding viral lysates following the cotransfection of the HIV-1 Gag-Pol construct and other expressing plasmids into HEK293T cells. Middle panel: Relative BST-2 reduction was determined by densitometric scanning using the ImageJ program (NIH). BST-2 level in cells that were cotransfected with HIV-1 Gag-Pol, BST-2 and pcDNA was arbitrarily set as 100%. Lower Panel: Relative HIV-1 VLPs release was assessed by densitometric scanning using the ImageJ program (NIH). The level of HIV-1 VLPs released from cells that were transfected with HIV-1 Gag-Pol and pcDNA was arbitrarily set as 100%. In both middle and lower panels of (a) and (b), the data shown were the average of three independent experiments. Click here for file
