# Supplementary figures and images for: Identification of novel key amino acids at the interface of the transmembrane domains of human BST-2 and HIV-1 Vpu
Source: Retrovirology. 2013 Aug 6;10:84. doi: 10.1186/1742-4690-10-84 (PMC3751551; doi:10.1186/1742-4690-10-84)

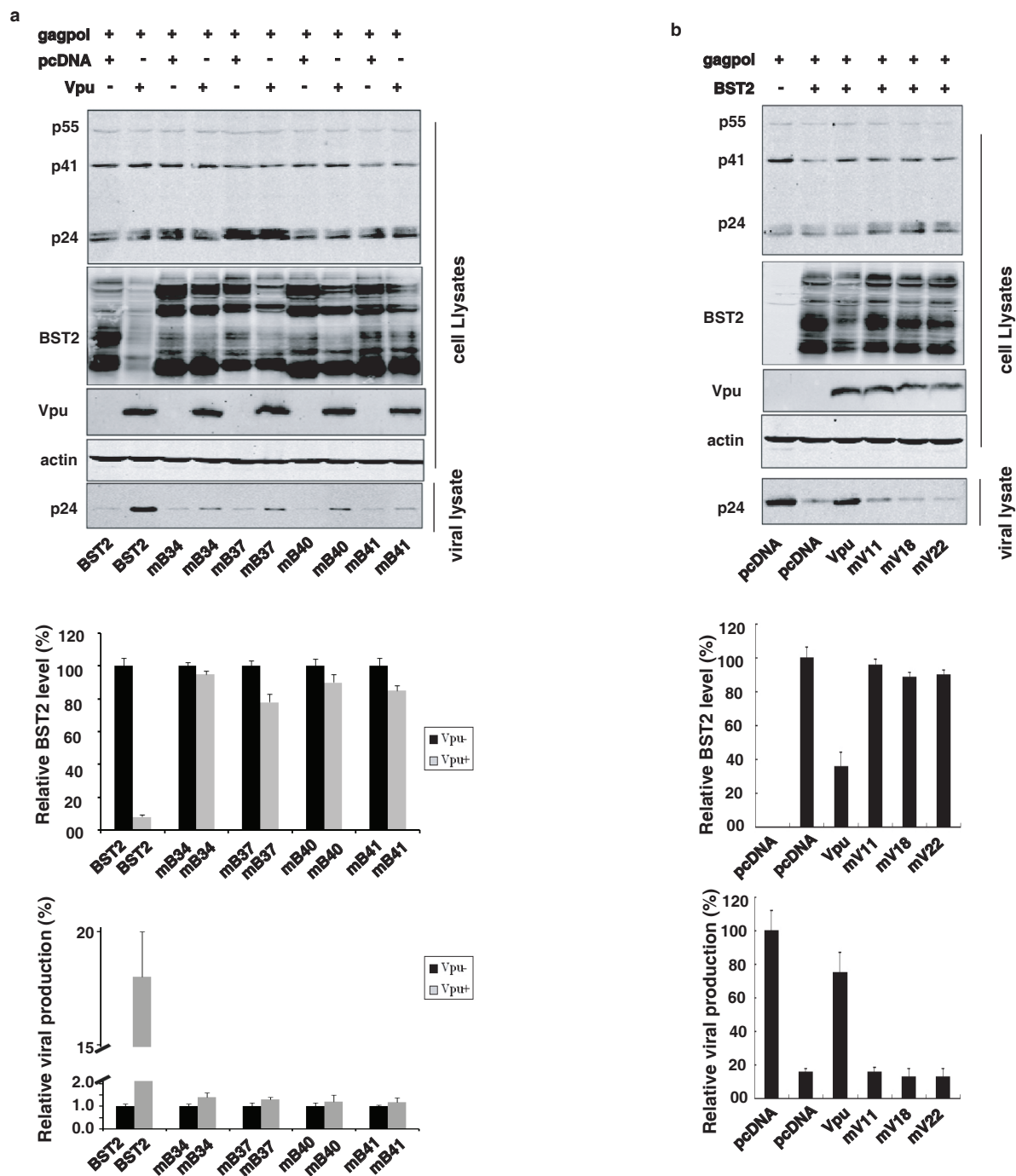

Figure S1

Supplement: Additional file 1: Figure S1 — Functional validation of the crucial residues in the TMD of BST-2 and Vpu for their interaction (a) Crucial residues in the TMD of BST-2 for Vpu interaction. Upper panel: Western blot analysis of the cellular lysates and the corresponding viral lysates following the cotransfection of the HIV-1 Gag-Pol construct and other plasmids into HEK293T cells. Middle panel: Relative BST-2 reduction was determined by densitometric scanning using the ImageJ program (NIH) aiming at the specific bands. BST-2 level in cells that were cotransfected with BST-2 or BST-2 mutants and together with pcDNA was arbitrarily set as 100%. Lower Panel: Relative HIV-1 VLPs release was assessed by densitometric scanning using the ImageJ program (NIH). The level of HIV-1 VLPs released from cells that were cotransfected of the HIV-1 Gag-Pol construct and BST-2 or BST-2 mutants and together with pcDNA was arbitrarily set as 100%. (b) Crucial residues in the TMD of Vpu for BST-2 interaction. Upper panel: Western blot analysis of the cellular lysates and the corresponding viral lysates following the cotransfection of the HIV-1 Gag-Pol construct and other expressing plasmids into HEK293T cells. Middle panel: Relative BST-2 reduction was determined by densitometric scanning using the ImageJ program (NIH). BST-2 level in cells that were cotransfected with HIV-1 Gag-Pol, BST-2 and pcDNA was arbitrarily set as 100%. Lower Panel: Relative HIV-1 VLPs release was assessed by densitometric scanning using the ImageJ program (NIH). The level of HIV-1 VLPs released from cells that were transfected with HIV-1 Gag-Pol and pcDNA was arbitrarily set as 100%. In both middle and lower panels of (a) and (b), the data shown were the average of three independent experiments. [file 1742-4690-10-84-S1.pdf]
